# Subcortical processing of speech regularities underlies reading and music aptitude in children

**DOI:** 10.1186/1744-9081-7-44

**Published:** 2011-10-17

**Authors:** Dana L Strait, Jane Hornickel, Nina Kraus

**Affiliations:** 1 Auditory Neuroscience Laboratory, Northwestern University, Evanston, IL, USA; 2 Institute for Neuroscience, Northwestern University, Chicago, IL, USA; 3 Department of Communication Sciences, Northwestern University, Evanston, IL, USA; 4 Department of Neurobiology and Physiology, Northwestern University, Evanston, IL, USA; 5 Department of Otolaryngology, Northwestern University, Chicago, IL, USA

## Abstract

**Background:**

Neural sensitivity to acoustic regularities supports fundamental human behaviors such as hearing in noise and reading. Although the failure to encode acoustic regularities in ongoing speech has been associated with language and literacy deficits, how auditory expertise, such as the expertise that is associated with musical skill, relates to the brainstem processing of speech regularities is unknown. An association between musical skill and neural sensitivity to acoustic regularities would not be surprising given the importance of repetition and regularity in music. Here, we aimed to define relationships between the subcortical processing of speech regularities, music aptitude, and reading abilities in children with and without reading impairment. We hypothesized that, in combination with auditory cognitive abilities, neural sensitivity to regularities in ongoing speech provides a common biological mechanism underlying the development of music and reading abilities.

**Methods:**

We assessed auditory working memory and attention, music aptitude, reading ability, and neural sensitivity to acoustic regularities in 42 school-aged children with a wide range of reading ability. Neural sensitivity to acoustic regularities was assessed by recording brainstem responses to the same speech sound presented in predictable and variable speech streams.

**Results:**

Through correlation analyses and structural equation modeling, we reveal that music aptitude and literacy both relate to the extent of subcortical adaptation to regularities in ongoing speech as well as with auditory working memory and attention. Relationships between music and speech processing are specifically driven by performance on a musical rhythm task, underscoring the importance of rhythmic regularity for both language and music.

**Conclusions:**

These data indicate common brain mechanisms underlying reading and music abilities that relate to how the nervous system responds to regularities in auditory input. Definition of common biological underpinnings for music and reading supports the usefulness of music for promoting child literacy, with the potential to improve reading remediation.

## 

The human nervous system makes use of sensory regularities to drive accurate perception, especially when confronted with challenging perceptual environments [1]. It is thought that the brain shapes perception according to predictions that are made based on regularities; this shaping is accomplished by comparing higher-level predictions with lower-level sensory encoding of an incoming stimulus via the corticofugal (i.e., top down) system [2]. This is a common neural feature that spans sensory modalities and can be observed in neural responses to regularly-occurring, as opposed to unpredictably-occurring, stimuli [3-5]. The brain's ability to use sensory regularities is a fundamental feature of auditory processing, promoting even the most basic of auditory experiences such as language processing during infancy [6,7] and speech comprehension amidst a competing conversational background [5]. Failure of the brain to utilize sensory regularities has been associated with neural dysfunction, such as schizophrenia [8] and language impairment (e.g., dyslexia) [5,9-11].

The impact of stimulus regularity on auditory processing has been well established in the auditory cortex [1,3] and was recently documented at and below the level of the brainstem [12-15]. Specifically, neural potentials to frequently-occurring sounds exhibit enhanced frequency tuning in both the primary auditory cortex [16] and in the auditory brainstem [5,17]. This sensory fine-tuning occurs rapidly, does not require overt attention and may enable enhanced object discrimination [14,18]. Although reference to the neural enhancement of a repeated speech sound might seem contradictory to the well-known repetition suppression of cortical evoked response magnitudes, the neural mechanisms underlying this effect remain debated. While some have proposed that stimulus repetition leads to overall decreased neuronal activity, others have suggested that repetition facilitates precision in neural representation by enhancing certain aspects of the neural response while inhibiting others (e.g., more precise inhibitory sidebands surrounding a facilitated response to the physical dimensions of a repeated stimulus) [4].

Human auditory brainstem responses (ABRs) to the pitch of predictably presented speech are enhanced relative to ABRs to speech presented in a variable context [5]. The extent of this subcortical enhancement of regularly-occurring speech relates to better performance on language-related tasks, such as reading and hearing speech in noise. This fine-tuning is thought to be driven by top-down cortical modulation of subcortical response properties [19] and its absence in poor readers is consistent with proposals that child reading impairment stems from the brain's inability to benefit from repetition in the sensory stream. Specifically, children with dyslexia fail to form perceptual anchors--a type of perceptual memory--based on repeating sounds [9,11].

Although we have made gains in understanding the auditory processing of speech regularities in children with reading *impairment *(or lack thereof), we do not know how auditory *expertise *shapes these mechanisms. The auditory expertise engendered by musical training during childhood and into adulthood promotes the subcortical encoding of speech [20,21] and may strengthen neural mechanisms that undergird child literacy [22-24]. Although the integrative nature of music and language abilities continues to be debated [25-27], a growing body of work supports shared abilities for music and reading, with music aptitude accounting for a substantial amount of the variance in child reading ability [28-30] even after controlling for nonverbal IQ and phonological awareness [31]. It is thought that strengthened top-down control, which is important for modulating lower-level neural responses, unfolds with expertise [32] and, more specifically, with musical training [33,34].

In order to define relationships between musical skill and literacy-related aspects of auditory brainstem function, we assessed subcortical processing of speech regularities, music aptitude and reading abilities in school-aged children. Our overarching goal was to define common biological underpinnings for music and reading abilities. We anticipated that music aptitude and literacy abilities would positively correlate with subcortical spectral enhancement of repetitive speech cues. We also explored relationships between musical skill and literacy-related aspects of auditory cognitive function through working memory assessments [35,36], which included an auditory attention component. We anticipated that music aptitude and literacy abilities would positively correlate with auditory working memory and attention performance. In order to delineate and quantify relationships among variables, we applied the data to Structural Equation Modeling (SEM). SEM relies on a variety of simultaneous statistical methods (e.g., factor analysis, multiple regressions and path analysis combined with structural equation relations) to evaluate a hypothesized model [37]. Although more traditional regression analyses are useful for delineating causal relationships among variables, SEM enables more efficient characterization of complex, real-world processes than can be achieved using correlation-based analyses [38]. Specific benefits of SEM include the simultaneous analysis of multiple interrelated variables, consideration of measurement error, and inherent control for multiple comparisons. We expected SEM to substantiate our hypothesis that music aptitude predicts much of the variance in literacy abilities by way of shared cognitive and neural mechanisms.

## Materials and methods

### Participants

42 normal hearing children between the ages of 8-13 years (M = 10.4, SD = 1.6, Males = 26). Participants and their legal guardians provided informed assent and consent according to Northwestern University's Institutional Review Board. Because we aimed to evaluate neural function and music aptitude across a spectrum of readers, no literacy restrictions were applied but all participants demonstrated normal audiometric thresholds (≤20 dB HL pure tone thresholds at octave frequencies from 125 to 8000 Hz) and IQ (≥85 score on the Wechsler Abbreviated Scale of Intelligence) [39]. Participants also had clinically normal ABRs to 80 dB SPL 100 μs click stimuli that were presented at 31.1 Hz.

Extent of extracurricular activity was assessed by a parent questionnaire (the Child Behavior Checklist [40]). Parents rated their child's current extracurricular activities according to the frequency of the child's involvement--less than average, average, or more than average; these scores were summed to produce a single extracurricular activity score.

Good (n = 8) and poor readers (n = 21) were differentiated based on reading ability (Test of Word Reading Efficiency; see *Reading and working memory*, below) [5]. Children with scores ≤90 were included in the poor reading group, while good readers had scores ≥110. 13 subjects did not meet the criteria for either group and were excluded from group analyses. Good and poor readers did not differ in age (Mann-Whitney *U *test; z = -0.223, p = 0.83), sex (Pearson Chi-Square χ^2 ^= 0.12, p = 0.73), socioeconomic status as inferred by maternal education [41] (Pearson Chi-Square χ^2 ^= 1.10, p = 0.59), years of musical training (Mann-Whitney *U *test; z = -0.231, p = 0.82), extent of extracurricular activity (Mann-Whitney *U *test; z = -1.202, p = 0.23) or nonverbal IQ (Mann-Whitney *U *test; z = -1.834, p = 0.07). With regard to musical training histories, 36 of the 42 children had undergone no to only a few months of musical training and were not currently involved in music activities. The other six children had participated in at least one year of musical training. One of these children was categorized as a poor reader, two were categorized as good readers and three were considered average readers (as such, these three were not included in either reading group).

### Reading and working memory

Standardized literacy measures assessed oral (Test of Word Reading Efficiency, TOWRE) [42] and silent (Test of Silent Word Reading Fluency, TOSWRF) [43] reading speed. The TOWRE requires children to read aloud lists of real words (Sight subtest) and nonsense words (Phonemic Decoding subtest) while being timed. The two subscores are combined to form a composite score (here referred to as the TOWRE). The TOSWRF requires participants to quickly identify printed words by demarcating lines of letters into individual words while being timed. Participants are presented with rows of words that gradually increase in reading difficulty and they are asked to separate them (e.g., *dimhowfigblue *→ *dim/how/fig/blue*). TOWRE ("reading efficiency") and TOSWRF ("reading fluency") age-normed scores were averaged in order to create a composite Reading variable for correlation analyses.

Auditory working memory was assessed using the Memory for Digits Forward subtest of the Comprehensive Test of Phonological Processing [44] and the Memory for Digits Reversed subtest of the Woodcock Johnson Test of Cognitive Abilities [45]. Digits forward and digits reversed age-normed scores were averaged in order to create a composite score for correlation analyses. In light of auditory attention's contribution to memory for digits forward [46], composite performance on both digits forward and reversed subtests is referred to as Auditory Working Memory and Attention (AWM/Attn).

### Music aptitude

Music aptitude was assessed using Edwin E. Gordon's *Intermediate Measures of Music Audiation *(IMMA) [47], which measures children's abilities to internalize musical sound and compare two sequentially presented sound patterns. Tonal aptitude was assessed by the Tonal subtest, in which participants are presented with 40 pairs of musical excerpts that do not differ rhythmically but may differ melodically. Rhythm aptitude was assessed by the Rhythm subtest, in which participants are presented with 40 pairs of short excerpts that do not differ melodically but may differ rhythmically. For both subtests, participants indicate whether the two excerpts in each pair are the same or different. The subtest scores are combined to generate a composite music aptitude score. The rhythm, tonal and composite scores are normed by academic grade in order to produce percentile rankings.

### Auditory brainstem measures

**Figure 1:**
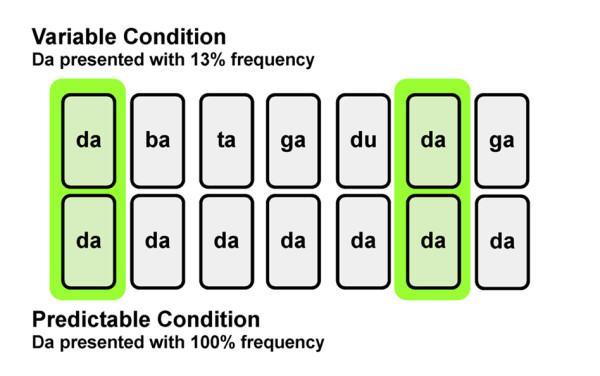
**Auditory brainstem response recording conditions. **We recorded ABRs to the same speech sound in two different conditions. For the *predictable *condition, /da/ was repeated at a probability of 100%. In the *variable *condition, /da/ was randomly interspersed in the context of seven other speech sounds. We trial-matched responses to compare ABRs recorded in the variable condition to those recorded in the predictable condition without the confound of presentation order or trial event.

Brainstem responses to the speech sound /da/ were collected from Cz using Scan 4.3 (Compumedics, Charlotte, NC) under two conditions. Ag-AgCl electrodes were applied in a vertical, ipsilateral montage (i.e., FPz as ground, right earlobe as reference). Evoked potentials recorded with this electrode montage have been found to reflect activity from an ensemble of neural elements of central brainstem origin [48,49]. In the predictable condition, the speech sound /da/ was presented at a probability of 100%, whereas in the variable condition /da/ was randomly interspersed in the context of seven other speech sounds at a probability of 13% (Figure 1). The seven speech sounds varied acoustically according to a variety of features, including formant structure (/ba/, /ga/, /du/), duration (a 163 ms /da/), voice-onset-time (/ta/) and F_0 _(250 Hz /da/, /da/ with a dipping pitch contour). The /da/ stimulus was a six-formant, 170 ms speech syllable synthesized in Klatt [50] with a 5 ms voice onset time and a level fundamental frequency (F_0_, 100 Hz). The first, second and third formants were dynamic over the first 50 ms (F_1_, 400-720 Hz; F_2_, 1700-1240 Hz; F_3_, 2580-2500 Hz) and then maintained frequency for the rest of the duration. The fourth, fifth and sixth formants were constant throughout the entire duration of the stimulus (F_4_, 3300 Hz; F_5_, 3750 Hz; F_6_, 4900 Hz). For a detailed description of the seven other speech sounds, see Chandrasekaran et al. (2009).

The stimulus was presented to the right ear via insert earphones (ER-3; Etymotic Research, Elk Grove Village, IL) at 80 dB SPL and at a rate of 4.35 Hz. This fast presentation rate limits the contribution of cortical neurons, which are unable to phase-lock at such fast rates [49]. Furthermore, the stimulus was presented in alternating polarities and average responses to each polarity were subsequently summed in order to limit contamination of the neural recording by the cochlear microphonic [51]. During recording sessions, participants watched videos of their choice in order to maintain a still yet wakeful state with the soundtrack quietly playing from a speaker, audible through the nontest ear. Because auditory input from the soundtrack was not stimulus-locked and stimuli were presented directly to the right ear at a +40 dB signal-to-noise ratio, the soundtrack had no significant impact on the recorded responses [51].

Responses were digitally sampled at 20,000 Hz, offline filtered from 70 to 2000 Hz with a 12 dB roll-off and epoched from -40 to 190 ms (stimulus onset at time zero). Events with amplitudes beyond ± 35 μV were rejected as artifacts. Responses to 100 μs clicks were collected before and after each recording session in order to ensure consistency of wave V latencies, confirming no differences in recording parameters or subject variables.

As in Chandrasekaran et al. [5], we compared the brainstem responses to /da/ recorded in the variable condition to trial-matched responses recorded to /da/ in the predictable condition (Figure 1). Specifically, neural responses in the predictable condition were averaged according to their occurrence relative to the order of presentation in the variable condition, resulting in 700 artifact-free responses for each condition.

In accordance with Chandrasekaran et al., we examined the strength of the spectral encoding of the second and fourth harmonics (H_2 _and H_4_) in average responses for each participant over the formant transition of the stimulus (7-60 ms in the neural response) via fast Fourier transforms executed in Matlab 7.5.0 (The Mathworks, Natick, MA). Spectral magnitudes were calculated for 10 Hz-wide bins surrounding H_2 _and H_4_. The differences in the spectral amplitudes of H_2 _and H_4 _between the two conditions (predictable minus variable) were calculated for each participant and normalized through conversion to a *z*-score based on the group mean.

### Statistical Analyses

The brainstem response *z*-scores were compared across conditions and groups using a Repeated Measures ANOVA and correlated with the reading and music aptitude measures using Pearson's correlations (SPSS Inc., Chicago, IL). RMANOVA outcomes were further defined in a post-hoc analysis using Mann-Whitney *U*-tests. All results reflect two-tailed values and normality for all data was confirmed using the Kolmogorov-Smirnov test for equality.

### Structural Equation Modeling

We normalized all data through conversion to *z*-scores based on group means. Analysis of covariance matrix structures was conducted with *Lisrel 8.8 *(Scientific Software International Inc., Lincolnwood, IL) and solutions were generated based on maximum-likelihood estimation. We defined the model's directions of causality in accordance with our aims, being to define common biological and cognitive factors to account for the covariance in child reading and music abilities. We selected the Root Mean Square Error of Approximation (RMSEA) in order to evaluate the model's goodness of fit, with measurements below 0.08 indicative of good model fit [52]. *Lisrel 8.8 *also calculates the likelihood ratio (χ^2^), its degrees of freedom and probability whenever maximum likelihood ratios are computed. The χ^2 ^test functions as a statistical method for evaluating structural models, describing and evaluating the residuals that result from fitting a model to the observed data. A χ^2 ^probability value greater than 0.05 indicates a good model fit [52].

## Results

The extent of subcortical enhancement of repetitive speech cues correlated with music aptitude and literacy abilities. Common variance among subcortical enhancement of repetitive speech cues, music aptitude and reading abilities was not accounted for by overarching factors such as socioeconomic status, extracurricular involvement or IQ.

SEM indicates that, by way of common neural (auditory brainstem) and cognitive (auditory working memory/attention) functions, music skill accounts for 38% of the variance in reading performance. The resulting statistical model delineates and quantifies relationships among auditory brainstem function, music aptitude, memory/attention and literacy.

### Music aptitude correlates with reading performance

Music aptitude correlated with reading performance. These relationships were largely driven by performance on the Rhythm music aptitude subtest (Rhythm-TOWRE: r = 0.41, p < 0.01; Rhythm-TOSWRF: r = 0.31, p < 0.05; Tonal-TOWRE: r = 0.16, p = 0.32; Tonal-TOSWRF: r = 0.26, p = 0.09), although the relationships between music aptitude and reading performance were strongest when considering the composite music aptitude score, which considers both Tonal and Rhythm performance (Composite-TOWRE: r = 0.45, p < 0.005; Composite-TOSWRF: r = 0.39, p < 0.01).

### Subcortical enhancement of predictable speech relates with reading and music abilities

**Figure 2:**
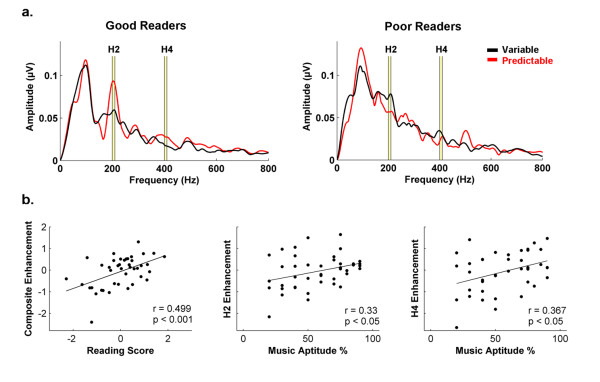
**Subcortical enhancement of predictable speech relates with music and reading abilities. (A) **Good readers demonstrate greater enhancement of speech presented in the predictable condition, compared to the variable condition, than poor readers. **(B) **The amount of enhancement observed in the predictable condition positively correlates with reading ability and music aptitude.

Poor readers showed weaker subcortical enhancement of spectral components of speech sounds (2^nd ^and 4^th ^harmonics) presented in the predictable, contrasted with the variable, condition than good readers (Figure 2a). No other significant neural differences were observed between groups, such as for the subcortical enhancement of the F_0 _or other harmonics. A 2 (condition) × 2 (reading group) × 2 (harmonic) RMANOVA demonstrated an interaction between condition and reading group (F = 13.33, p < 0.001). Post-hoc Mann Whitney *U*-tests demonstrated that good readers have a greater enhancement of speech harmonics presented in the predictable condition than poor readers (H_2_: z = -2.25, p < 0.05; H_4_: z = -2.98, p < 0.005; Figure 2a).

The amount of enhancement observed in ABRs recorded in the predictable compared to the variable condition positively correlated with reading and music aptitude performance across all subjects. The reading composite score (produced by combining TOWRE and TOSWRF z-scores) correlated with the amount of brainstem enhancement for both H_2 _and H_4 _(H_2_: r = 0.44, p < 0.005; H_4_: r = 0.40, p < 0.01; Figure 2b). The music composite score also correlated with the amount of brainstem enhancement to both harmonics (H_2_: r = 0.33, p < 0.05; H_4_: r = 0.37, p < 0.01; Figure 2b).

### Auditory working memory and attention relate with reading and music abilities

**Figure 3:**
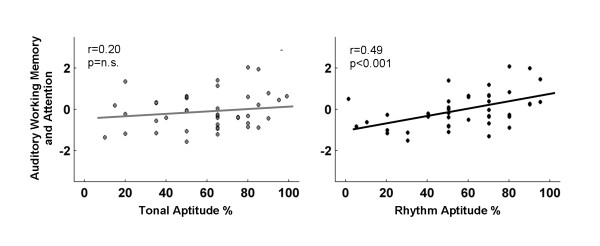
**Auditory working memory correlates with music aptitude. **Higher rhythm, but not tonal, aptitude correlates with better auditory working memory and attention (AWM/Attn) performance.

Reading and music aptitude positively correlated with performance on the auditory working memory tasks--memory for digits forward and digits reversed. Higher AWM/Attn correlated with better reading performance (TOWRE: r = 0.45, p < 0.005; TOSWRF: r = 0.38, p < 0.01). Likewise, higher AWM/Attn correlated with higher music aptitude (r = 0.44, p < 0.005). The relationship between AWM/Attn and music aptitude appeared to be largely driven by the rhythm subtest (Tonal: r = 0.203, p < 0.20; Rhythm: r = 0.49, p < 0.001; Figure 3).

Although AWM/Attn correlated with the amount of brainstem enhancement to both harmonics (r = 0.35, p < 0.05), the covariance between these measures could be accounted for by their relationships with music aptitude. Whereas partialing for AWM/Attn did not eliminate the common variance observed between music aptitude and repetitive harmonic enhancement (r = 0.32, p = 0.04), AWM/Attn and repetitive harmonic enhancement no longer covaried when partialing for music aptitude (r = 0.20, p = 0.20). This suggests that most of the covariance between AWM/Attn and repetitive harmonic enhancement can be explained by their shared variance with music aptitude.

### Consideration of overarching factors

Common variance among subcortical enhancement of repetitive speech cues, music aptitude and reading abilities could not be accounted for by overarching factors such as IQ, socioeconomic status (SES) or extracurricular involvement (ExCurr). SES and ExCurr did not correlate with any of our observed variables (Table 1). IQ, on the other hand, accounted for a significant amount of the variance in our test variables (brainstem function: r = 0.37, p < 0.02; reading performance: r = 0.45, p < 0.02; auditory working memory: r = 0.37, p < 0.001). Although IQ did not correlate with overall music aptitude or the tonal aptitude subscore (composite: r = 0.25, p = 0.11; tonal: r = 0.02, p = 0.89), it correlated with the rhythm aptitude subscore (r = 0.38, p < 0.02). Given that covarying for IQ did not eliminate the correlations observed among our test variables (music × reading: r = 0.41, p = 0.03; music × memory/attention: r = 0.47, p = 0.01; music × subcortical function: r = 0.41, p = 0.03; reading × subcortical function: r = 0.52, p = 0.004; reading × memory/attention: r = 0.43, p = 0.04), we conclude that IQ did not account for the common variance reported among music aptitude, reading ability, working memory/attention and subcortical and cognitive function.

**Table 1:** Subjects' socioeconomic status (SES) and extracurricular activity involvement did not correlate with the test variables of music aptitude, auditory brainstem enhancement of repetitive speech cues, reading, or auditory memory/attention

	SES	ExCurr
**Music aptitude**	-0.06, 0.72	0.02, 0.90

**Brainstem function**	0.19, 0.26	0.02, 0.88

**Reading**	-0.04, 0.80	0.16, 0.31

**Auditory memory/attention**	-0.01, 0.93	0.12, 0.45

Table input represents Pearson's *r *and *p *values.

### Modeling relationships among music aptitude, reading ability and subcortical function

**Figure 4:**
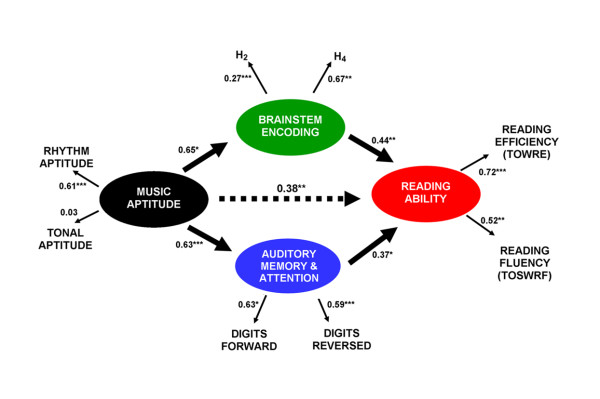
**Structural equation model (SEM) of music aptitude, reading, auditory working memory/attention and auditory brainstem function. **Music aptitude accounts for 38% of the variability in reading ability through its impact on auditory working memory/attention and subcortical enhancement of predictable speech harmonics. The model demonstrates an excellent fit; values plotted represent squared correlation coefficients (*r*^2^). *p < 0.05; **p < 0.01; ***p < 0.001.

In order to more comprehensively examine relationships among music aptitude, subcortical processing of speech regularities and reading ability, we subjected these data to SEM [37]. SEM provides a mathematical method for evaluating relationships among independent and dependent variables in a model hypothesized *a priori*. Our hypothesized model, depicted in Figure 4, projected that music aptitude predicts reading ability by means of subcortical processing of speech regularities and AWM/Attn function.

By means of subcortical enhancement of predictable speech harmonics and AWM, music aptitude accounted for 38% of the variability in reading ability (p < 0.01). The model demonstrated an excellent fit (χ^2^(_18_) = 17.64, p > 0.35; RMSEA = 0.05). All path coefficients were significant except for the path between Tonal Aptitude and Composite Music Aptitude (r^2 ^= 0.03, p = 0.31). This model emphasizes the combined strength of relationships among rhythm aptitude, subcortical enhancement of predictable speech harmonics and AWM/Attn in predicting child reading ability.

## Discussion

We observed correlations among music and literacy abilities with the extent of subcortical enhancement of predictable speech cues. As such, our data reveal common, objective neural markers for music aptitude and reading ability and suggest a model for the relationships that have been documented between music and literacy performance [28-31,53].

Our data also reveal common cognitive markers for music aptitude and reading ability. Auditory working memory and attention are driving components of child literacy [35,36], and relationships between auditory working memory and attention and musical skill have already been established [33,54]. Not only do musicians demonstrate better verbal memory than nonmusicians, but this advantage can be seen with as little as one year of musical training [55]. Our results demonstrate a similar relationship between auditory working memory and attention and music aptitude in children, although this relationship is observed regardless of musical training backgrounds.

### The role of the descending auditory system

As in Chandrasekaran et al., we observed subcortical enhancement of a predictable, contrasted with a variable, speech presentation [5]. This enhancement was specific for frequencies integral to the perception of pitch (H_2 _and H_4_). Similar repetition-induced frequency enhancement has been observed in the primary auditory cortex, where neurons exhibit sharpened acuity to stimulus frequency [16]. This tuning occurs without overt attention, is stimulus specific and develops rapidly [3,56]. Not surprisingly, enhanced neural tuning with stimulus repetition has been proposed to relate with improved object discrimination [16,18].

The ability of the sensory system to automatically modify neural response properties according to expectations in a dynamic and context-sensitive manner is thought to have evolved to infer and represent the causes of change in our environment [1,57]. This modification may occur in a descending fashion, beginning in extra-sensory cortices where predictions are developed based on prior experience (such as with repetition) and sequentially tuning lower level response properties to heighten sensory acuity [2,32,57,58]. The descending nature of this neural tuning is supported by observations from cortical work showing decreased onset latencies from 120 ms (after two repetitions) to 50 ms (after 30 repetitions) [56] and is thought to represent the strengthening of the stimulus-specific memory trace at earlier and earlier processing stages [3]. The correlations reported here between music aptitude and reading ability with subcortical fine-tuning to predictable speech sounds may indicate stronger top-down modulatory systems in individuals with better music aptitude and reading performance.

### Musical experience boosts sensitivity to sound patterns

Our data demonstrate that diminished subcortical enhancement of predictable speech sounds relates with reading impairment. Similar observations have been made in poor readers, in addition to children with poor perception of speech presented in background noise [5]; we extend these findings to the domain of music. This relationship is not surprising given the importance of sound repetition and sequencing for music perception. Specifically, repetition and regularity lends to the perception of tonality [59], rhythm and meter [60,61] and the structural use of musical themes. Deviations from predicted patterns result in impaired music production and perception [62-64] and can be flagged by the auditory cortex in both musically trained and untrained individuals, as measured by auditory evoked potentials [65-67]. Increased sensitivity to deviations from patterns in musical sound is thought to reflect enhanced sensory memory and discrimination abilities as well as more firmly established categorical boundaries [68].

It is not surprising that we observed correlations between music aptitude and subcortical spectral enhancement of predictable speech sounds given that musical expertise increases one's sensitivity to sound patterns not only in music, but also in speech [34,69]. Although the argument can be made for a genetic contributor to musicians' enhanced sound processing, this increased sensitivity can be modulated, at least in part, by one's method of musical practice and training [70]. Furthermore, diverse methodological approaches consistently reveal correlations between the extent of structural and functional neural enhancement observed in musicians and their years of musical practice or age of practice onset [71-74]. Such observations suggest the substantial contribution of experience-induced neuroplasticity to musicians' enhanced sound processing and may be attributed to the strength of top-down contributors to auditory processing [33,69].

### Subcortical enhancement of predictable speech: implications for reading impairment

Due to its multisensory nature, attentional demands and reliance on rapid audio-motor feedback, music is a powerful tool for engendering neural plasticity, particularly for auditory processing [34,75-78]. This plasticity is not constrained to the brain's music networks but applies more generally to auditory functions [27,69,72,79-82]. Clinicians and researchers involved in the treatment and assessment of reading dysfunction have long held interest in the potential for musical training to strengthen neural networks for reading. Wisbey was one of the first to formally propose that music, by facilitating the development of multisensory awareness and auditory acuity, could promote reading in impaired children [83]. This proposal has been verified by a number of experiments [84,85] (c.f. Morais *et al*., 2010 [25]), with relationships between music and reading abilities observed in many more [28-30,53,86].

Definition and characterization of common neural mechanisms for music and reading skills may enable the development of a biological assessment of reading impairment and improve the efficacy of remedial attempts. Reading performance is known to rely on a chorus of multifaceted and complex processes that have proven difficult to disentangle; here, we find that subcortical function serves as a significant and accessible factor in reading impairment, accounting for 44% of the variance in child reading ability. The use of auditory brainstem measurements to assess learning and reading impairment has emerged in recent years [21,87,88], is being adapted for the clinic and can provide an objective index of the success of auditory [89,90] and music training [21]. In light of the high test-retest reliability of the speech-evoked ABR [91], individual responses are highly replicable and can be meaningfully compared to group means or established norms. Identification of common neural markers for music and reading skill, such as those reported here, may lead to the biological assessment of music-associated learning abilities in children and encourage the employment of music as a technique for literacy remediation.

Musical training during early childhood may be particularly important for the advancement of music and reading aptitude. Although the music test employed here is thought to measure music aptitude, being one's inherent ability for music, the creator of this measure, Edwin E. Gordon, has long emphasized the impact of music education during early childhood on music aptitude scores. Gordon makes this claim in light of his extensive longitudinal work showing that music aptitude can improve with musical training, particularly during early childhood [92]. The importance of an early onset of music activities is more directly supported by outcomes from neuroscientific research, in which many of the neuroplastic changes associated with musical training are more extensive in individuals who began training earlier in their lifetimes [71,72,93-96]. With regard to auditory brainstem processing, we found that ABRs in young adult musicians who began musical training prior to age 7 were distinct from those in musicians who began training between the ages of 7-13 [72,93]. Whereas musicians who began training prior to age 7 demonstrated enhanced ABRs to the spectral components of communication sounds compared to nonmusicians, those who began later in life did not. Observations such as this reflect a critical period for musical training-associated neural plasticity [97] and may speak to the importance of initiating musical training during early childhood for bringing about the greatest impact on music aptitude or, we propose, reading ability.

It remains undetermined whether reading abilities are impacted alongside music aptitude with musical training during childhood or whether the neural mechanism reported here is affected by musical training. Also undetermined is whether relationships between music and reading work in reverse, with language-based literacy remediation leading to improved music aptitude. More work (notably, longitudinal work) is necessary in order to define relationships between music aptitude, literacy and the auditory brainstem response to speech as well as to determine the impact of formal training, the efficacy of specific training approaches and/or literacy remediation programs.

### Conclusions

Reading relies on a complex and multifaceted combination of processes that have proven difficult to disentangle. In light of correlational and structural modeling analyses, we conclude that subcortical function serves as a significant and accessible factor underlying reading ability and impairment, predicting 44% of the variance in reading ability. Further outcomes reveal direct relationships between musical skill and literacy-related aspects of auditory brainstem and memory/attention function, revealing common neural and cognitive mechanisms for reading and music abilities that may operate, at least in part, via corticofugal shaping of sensory function. By way of auditory brainstem spectral enhancement of predictable speech and auditory working memory/attention, music skill predicts approximately 40% of the variance in reading performance. Definition of common neural and cognitive mechanisms for music and reading skills may support the usefulness of music for promoting child literacy, with the potential to improve the efficacy of remedial attempts.

## Competing interests

The authors declare that they have no competing interests.

## Authors' contributions

DS collected the data, conducted and interpreted the statistical analyses and prepared the manuscript. JH collected and processed the data, provided consultation with respect to statistical methods and reviewed the drafts of the manuscript. NK oversaw all aspects of the study and reviewed the drafts of the manuscript. All authors read and approved the final manuscript.

## Appendix A

### Grouping according to good and poor music aptitude

The extent of brainstem enhancement of predictable speech in subjects with high (IMMA ≥70^th ^percentile; n = 18) and low (IMMA ≤30^th ^percentile; n = 9) music aptitude patterned with the results observed when subjects were divided into good and poor readers. A 2 (condition) × 2 (music group) × 2 (harmonic) RMANOVA demonstrated an interaction between condition and music group (F = 6.17, p < 0.02). Post-hoc Mann Whitney *U*-tests demonstrated that subjects with high music aptitude have a greater enhancement of the second harmonic of speech presented in the predictable condition compared to the variable condition than subjects with low music aptitude (H_2_: z = -1.96, p < 0.05; H_4_: z = -1.29, p = 0.19).

## References

[B1] Winkler I Denham SL Nelken I Modeling the auditory scene: Predictive regularity representations and perceptual objects Trends Cogn Sci 2009 13 532 40 10.1016/j.tics.2009.09.003 19828357

[B2] Ahissar M Nahum M Nelken I Hochstein S Reverse hierarchies and sensory learning Philos Trans R Soc Lond B Biol Sci 2009 364 285 99 10.1098/rstb.2008.0253 18986968 PMC2674477

[B3] Baldeweg T Repetition effects to sounds: Evidence for predictive coding in the auditory system Trends Cogn Sci 2006 10 93 4 10.1016/j.tics.2006.01.010 16460994

[B4] Grill-Spector K Henson R Martin A Repetition and the brain: Neural models of stimulus-specific effects Trends Cogn Sci 2006 10 14 23 10.1016/j.tics.2005.11.006 16321563

[B5] Chandrasekaran B Hornickel J Skoe E Nicol T Kraus N Context-dependent encoding in the human auditory brainstem relates to hearing speech in noise: Implications for developmental dyslexia Neuron 2009 64 311 9 10.1016/j.neuron.2009.10.006 19914180 PMC2778610

[B6] Pelucchi B Hay JF Saffran JR Statistical learning in a natural language by 8-month-old infants Child Dev 2009 80 674 85 10.1111/j.1467-8624.2009.01290.x 19489896 PMC3883431

[B7] Saffran JR Aslin RN Newport EL Statistical learning by 8-month-old infants Science 1996 274 1926 8 10.1126/science.274.5294.1926 8943209

[B8] Stephan KE Baldeweg T Friston KJ Synaptic plasticity and dysconnection in schizophrenia Biol Psychiatry 2006 59 929 39 10.1016/j.biopsych.2005.10.005 16427028

[B9] Ahissar M Lubin Y Putter-Katz H Banai K Dyslexia and the failure to form a perceptual anchor Nat Neurosci 2006 9 1558 64 10.1038/nn1800 17115044

[B10] Schulte-Korne G Deimel W Bartling J Remschmidt H Pre-attentive processing of auditory patterns in dyslexic human subjects Neurosci Lett 1999 276 41 4 10.1016/S0304-3940(99)00785-5 10586970

[B11] Evans JL Saffran JR Robe-Torres K Statistical learning in children with specific language impairment J Speech Lang Hear Res 2009 52 321 35 10.1044/1092-4388(2009/07-0189) 19339700 PMC3864761

[B12] Malmierca MS Cristaudo S Perez-Gonzalez D Covey E Stimulus-specific adaptation in the inferior colliculus of the anesthetized rat J Neurosci 2009 29 5483 93 10.1523/JNEUROSCI.4153-08.2009 19403816 PMC2715893

[B13] Dean I Robinson BL Harper NS McAlpine D Rapid neural adaptation to sound level statistics J Neurosci 2008 28 6430 8 10.1523/JNEUROSCI.0470-08.2008 18562614 PMC6670892

[B14] Pressnitzer D Sayles M Micheyl C Winter IM Perceptual organization of sound begins in the auditory periphery Curr Biol 2008 18 1124 8 10.1016/j.cub.2008.06.053 18656355 PMC2559912

[B15] Wen B Wang GI Dean I Delgutte B Dynamic range adaptation to sound level statistics in the auditory nerve J Neurosci 2009 29 13797 808 10.1523/JNEUROSCI.5610-08.2009 19889991 PMC2774902

[B16] Ulanovsky N Las L Nelken I Processing of low-probability sounds by cortical neurons Nat Neurosci 2003 6 391 8 10.1038/nn1032 12652303

[B17] Dean I Harper NS McAlpine D Neural population coding of sound level adapts to stimulus statistics Nat Neurosci 2005 8 1684 9 10.1038/nn1541 16286934

[B18] Muller JR Metha AB Krauskopf J Lennie P Rapid adaptation in visual cortex to the structure of images Science 1999 285 1405 8 10.1126/science.285.5432.1405 10464100

[B19] Suga N Role of corticofugal feedback in hearing J Comp Physiol A Neuroethol Sens Neural Behav Physiol 2008 194 169 83 10.1007/s00359-007-0274-2 18228080

[B20] Bidelman GM Gandour JT Krishnan A Cross-domain effects of music and language experience on the representation of pitch in the human auditory brainstem J Cogn Neurosci 2009 23 425 434 19925180 10.1162/jocn.2009.21362

[B21] Kraus N Skoe E Parbery-Clark A Ashley R Experience-induced malleability in neural encoding of pitch, timbre, and timing Ann NY Acad Sci 2009 1169 543 57 10.1111/j.1749-6632.2009.04549.x 19673837 PMC2810198

[B22] Gaab N Tallal P Kim H Lakshminarayanan K Archie JJ Glover GH Gabrieli JD Neural correlates of rapid spectrotemporal processing in musicians and nonmusicians Ann NY Acad Sci 2005 1060 82 8 10.1196/annals.1360.040 16597753

[B23] Besson M Schon D Moreno S Santos A Magne C Influence of musical expertise and musical training on pitch processing in music and language Restor Neurol Neurosci 2007 25 399 410 17943015

[B24] Chandrasekaran B Kraus N Music, noise-exclusion, and learning Music Percept 2010 27 297 306 10.1525/mp.2010.27.4.297

[B25] Morais J Periot A Lidji P Kolinsky R Music and dyslexia Int J Arts Technolog 2010 3 177 194 10.1504/IJART.2010.032563

[B26] Zatorre RJ Gandour JT Neural specializations for speech and pitch: Moving beyond the dichotomies Philos Trans R Soc Lond B Biol Sci 2008 363 1087 104 10.1098/rstb.2007.2161 17890188 PMC2606798

[B27] Patel AD Why would musical training benefit the neural encoding of speech? The opera hypothesis Front Psychol 2011 2 142 21747773 10.3389/fpsyg.2011.00142 PMC3128244

[B28] Forgeard M Schlaug G Norton A Rosam C Iyengar U The relation between music and phonological processing in normal-reading children and children with dyslexia Music Percept 2008 25 383 390 10.1525/mp.2008.25.4.383

[B29] Overy K Dyslexia and music. From timing deficits to musical intervention Ann NY Acad Sci 2003 999 497 505 10.1196/annals.1284.060 14681173

[B30] Huss M Verney JP Fosker T Mead N Goswami U Music, rhythm, rise time perception and developmental dyslexia: Perception of musical meter predicts reading and phonology Cortex 2010 10.1016/j.cortex.2010.07.010 20843509

[B31] Anvari SH Trainor LJ Woodside J Levy BA Relations among musical skills, phonological processing, and early reading ability in preschool children J Exp Child Psychol 2002 83 111 30 10.1016/S0022-0965(02)00124-8 12408958

[B32] Ahissar M Hochstein S The reverse hierarchy theory of visual perceptual learning Trends Cogn Sci 2004 8 457 64 10.1016/j.tics.2004.08.011 15450510

[B33] Strait DL Kraus N Parbery-Clark A Ashley R Musical experience shapes top-down auditory mechanisms: Evidence from masking and auditory attention performance Hear Res 2010 261 22 29 10.1016/j.heares.2009.12.021 20018234

[B34] Kraus N Chandrasekaran B Music training for the development of auditory skills Nat Rev Neurosci 2010 11 599 605 20648064 10.1038/nrn2882

[B35] Share D Jorm A Maclean R Matthews R Sources of individual differences in reading acquisition J Educat Psycholog 1984 76 1309 1324

[B36] Jorm A Share D Maclean R Matthews R Cognitive factors at school entry predictive of specific reading retardation and general reading backwardness: A research note J Child Psychol Psychiatry 1986 27 45 54 10.1111/j.1469-7610.1986.tb00620.x 3949906

[B37] Jöreskog KG Lisrel 8: User's reference guide 1996 Scientific Software International, Inc.: Lincolnwood, IL18641497

[B38] Gefen D Straub D Boudreau MC Structural equation modeling and regression: Guidelines for research practice Communications of AIS 2000 4 1 80

[B39] Wechsler D Wechsler Abbreviated Scale of Intelligence (WASI) 1999 San Antonio, TX: Harcourt Assessment

[B40] Achenbach TM Ruffle TM The child behavior checklist and related forms for assessing behavioral/emotional problems and competencies Pediatr Rev 2000 21 265 71 10.1542/pir.21-8-265 10922023

[B41] Baydar N Brooks-Gunn J Furstenberg FF Early warning signs of functional illiteracy: Predictors in childhood and adolescence Child Dev 1993 64 815 29 10.2307/1131220 8339697

[B42] Torgeson JK Wagner RK Rashotte CA Test of Word Reading Efficiency 1999 Austin, TX: Pro-Ed

[B43] Mather N Hammill DD Allen EA Roberts R Test of Silent Word Reading Fluency 2004 Austin, TX: Pro-Ed

[B44] Wagner R Torgesen JK Rashotte C Ctopp: Comprehensive Test of Phonological Processing 1999 Austin, TX: Pro-ed

[B45] Woodcock RW McGre KS Mather N Woodcock-Johnson Psycho-educational Battery 2001 3 Itasca, IL: Riverside

[B46] Baddeley A Working memory: Looking back and looking forward Nat Rev Neurosci 2003 4 829 39 14523382 10.1038/nrn1201

[B47] Gordon EE Intermediate Measures of Music Audiation 1986 Chicago: GIA Publications, Inc

[B48] Galbraith GC Threadgill MR Hemsley J Salour K Songdej N Ton J Cheung L Putative measure of peripheral and brainstem frequency-following in humans Neurosci Lett 2000 292 123 7 10.1016/S0304-3940(00)01436-1 10998564

[B49] Chandrasekaran B Kraus N The scalp-recorded brainstem response to speech: Neural origins and plasticity Psychophysiology 2010 47 236 246 10.1111/j.1469-8986.2009.00928.x 19824950 PMC3088516

[B50] Klatt D Software for a cascade/parallel formant synthesizer J Acoust Soc Amer 1980 67 13 33

[B51] Skoe E Kraus N Auditory brain stem response to complex sounds: A tutorial Ear Hear 2010 31 10.1097/AUD.0b013e3181cdb272 PMC2868335 20084007

[B52] Hu L Bentler PM Fit indices in covariance structure modeling: Sensitivity to underparameterized model misspecification Psycholog Method 1998 3 424 453

[B53] Goswami U A temporal sampling framework for developmental dyslexia Trends Cogn Sci 2011 15 3 10 10.1016/j.tics.2010.10.001 21093350

[B54] Chan AS Ho YC Cheung MC Music training improves verbal memory Nature 1998 396 128 10.1038/24075 9823892

[B55] Ho YC Cheung MC Chan AS Music training improves verbal but not visual memory: Cross-sectional and longitudinal explorations in children Neuropsycholog 2003 17 439 450 10.1037/0894-4105.17.3.439 12959510

[B56] Haenschel C Vernon DJ Dwivedi P Gruzelier JH Baldeweg T Event-related brain potential correlates of human auditory sensory memory-trace formation J Neurosci 2005 25 10494 501 10.1523/JNEUROSCI.1227-05.2005 16280587 PMC6725828

[B57] Friston K A theory of cortical responses Philos Trans R Soc Lond B Biol Sci 2005 360 815 36 10.1098/rstb.2005.1622 15937014 PMC1569488

[B58] Bajo VM Nodal FR Moore DR King AJ The descending corticocollicular pathway mediates learning-induced auditory plasticity Nat Neurosci 2010 13 253 60 10.1038/nn.2466 20037578 PMC3634157

[B59] Krumhansl CL Perceiving tonal structure in music Amer Scient 1985 73 371 378

[B60] Hannon EE Snyder JS Eerola T Krumhansl CL The role of melodic and temporal cues in perceiving musical meter J Exp Psychol Hum Percept Perform 2004 30 956 74 15462633 10.1037/0096-1523.30.5.956

[B61] Large EW Jones MR The dynamics of attending: How people track time-varying events Psycholog Rev 1999 106 119 159

[B62] Repp BH London J Keller PE Production and synchronization of uneven rhythms at fast tempi Music Percept 2005 23 61 78 10.1525/mp.2005.23.1.61

[B63] Snyder JS Hannon EE Large EW Christiansen MH Synchronization and continuation tapping to complex meters Music Percept 2006 24 135 146 10.1525/mp.2006.24.2.135

[B64] Jones MR Moynihan H MacKenzie N Puente J Temporal aspects of stimulus-driven attending in dynamic arrays Psycholog Sci 2002 13 313 319 10.1111/1467-9280.00458 12137133

[B65] Koelsch S Siebel WA Towards a neural basis of music perception Trends Cogn Sci 2005 9 578 584 10.1016/j.tics.2005.10.001 16271503

[B66] Vuust P Ostergaard L Pallesen KJ Bailey C Roepstorff A Predictive coding of music - brain responses to rhythmic incongruity Cortex 2009 45 80 92 10.1016/j.cortex.2008.05.014 19054506

[B67] Trainor LJ McDonald KL Alain C Automatic and controlled processing of melodic contour and interval information measured by electrical brain activity J Cogn Neurosci 2002 14 430 442 10.1162/089892902317361949 11970802

[B68] Koelsch S Schroger E Tervaniemi M Superior pre-attentive auditory processing in musicians Neuroreport 1999 10 1309 1313 10.1097/00001756-199904260-00029 10363945

[B69] Tervaniemi M Kruck S De Baene W Schroger E Alter K Friederici AD Top-down modulation of auditory processing: Effects of sound context, musical expertise and attentional focus Eur J Neurosci 2009 30 1636 42 10.1111/j.1460-9568.2009.06955.x 19821835

[B70] Seppanen M Brattico E Tervaniemi M Practice strategies of musicians modulate neural processing and the learning of sound-patterns Neurobiol Learn Mem 2007 87 236 47 10.1016/j.nlm.2006.08.011 17046293

[B71] Wong PC Skoe E Russo NM Dees T Kraus N Musical experience shapes human brainstem encoding of linguistic pitch patterns Nat Neurosci 2007 10 420 2 17351633 10.1038/nn1872 PMC4508274

[B72] Strait DL Kraus N Skoe E Ashley R Musical experience and neural efficiency: Effects of training on subcortical processing of vocal expressions of emotion Eur J Neurosci 2009 29 661 8 10.1111/j.1460-9568.2009.06617.x 19222564

[B73] Gaser C Schlaug G Brain structures differ between musicians and nonmusicians J Neurosci 2003 23 9240 5 14534258 10.1523/JNEUROSCI.23-27-09240.2003 PMC6740845

[B74] Hutchinson S Lee LH Gaab N Schlaug G Cerebellar volume of musicians Cereb Cortex 2003 13 943 9 10.1093/cercor/13.9.943 12902393

[B75] Norton A Winner E Cronin K Overy K Lee DJ Schlaug G Are there pre-existing neural, cognitive, or motoric markers for musical ability? Brain Cogn 2005 59 124 34 10.1016/j.bandc.2005.05.009 16054741

[B76] Schlaug G The brain of musicians. A model for functional and structural adaptation Ann NY Acad Sci 2001 930 281 99 11458836

[B77] Schlaug G Forgeard M Zhu L Norton A Winner E Training-induced neuroplasticity in young children Ann NY Acad Sci 2009 1169 205 8 10.1111/j.1749-6632.2009.04842.x 19673782 PMC3005566

[B78] Schlaug G Norton A Overy K Winner E Effects of music training on the child's brain and cognitive development Ann NY Acad Sci 2005 1060 219 30 10.1196/annals.1360.015 16597769

[B79] Strait DL Kraus N Playing music for a smarter ear: Cognitive, perceptual and neurobiological evidence Music Percept in press 10.1525/MP.2011.29.2.133 PMC3444167 22993456

[B80] Musacchia G Sams M Skoe E Kraus N Musicians have enhanced subcortical auditory and audiovisual processing of speech and music Proc Natl Acad Sci USA 2007 104 15894 8 10.1073/pnas.0701498104 17898180 PMC2000431

[B81] Parbery-Clark A Skoe E Kraus N Musical experience limits the degradative effects of background noise on the neural processing of sound J Neurosci 2009 29 14100 7 10.1523/JNEUROSCI.3256-09.2009 19906958 PMC6665054

[B82] Schon D Magne C Besson M The music of speech: Music training facilitates pitch processing in both music and language Psychophysiology 2004 41 341 9 10.1111/1469-8986.00172.x 15102118

[B83] Wisbey AS Music as the source of learning 1980 Lancaster: M.T.P. Press, Ltd

[B84] Douglas S Willatts P The relationship between musical ability and literacy skills J Res Reading 1994 17 99 107 10.1111/j.1467-9817.1994.tb00057.x

[B85] Moreno S Marques C Santos A Santos M Castro SL Besson M Musical training influences linguistic abilities in 8-year-old children: More evidence for brain plasticity Cereb Cortex 2009 19 712 23 10.1093/cercor/bhn120 18832336

[B86] Overy K Nicolson RI Fawcett AJ Clarke EF Dyslexia and music: Measuring musical timing skills Dyslexia 2003 9 18 36 10.1002/dys.233 12625374

[B87] Banai K Hornickel J Skoe E Nicol T Zecker S Kraus N Reading and subcortical auditory function Cereb Cortex 2009 19 2699 707 10.1093/cercor/bhp024 19293398 PMC2758683

[B88] Hornickel J Skoe E Nicol T Zecker S Kraus N Subcortical differentiation of voiced stop consonants: Relationships to reading and speech in noise perception Proc Natl Acad Sci USA 2009 106 13022 13027 10.1073/pnas.0901123106 19617560 PMC2722305

[B89] Russo NM Nicol TG Zecker SG Hayes EA Kraus N Auditory training improves neural timing in the human brainstem Behav Brain Res 2005 156 95 103 10.1016/j.bbr.2004.05.012 15474654

[B90] Song JH Skoe E Wong PC Kraus N Plasticity in the adult human auditory brainstem following short-term linguistic training J Cogn Neurosci 2008 20 1892 902 10.1162/jocn.2008.20131 18370594 PMC2829864

[B91] Song JH Nicol T Kraus N Test-retest reliability of the speech-evoked auditory brainstem response Clin Neurophysiol 2010 10.1016/j.clinph.2010.07.009 PMC2990784 20719558

[B92] Gordon EE Tonal and rhythm patterns, an objective analysis: A taxonomy of tonal patterns and rhythm patterns and seminal experimental evidence of their difficulty and growth rate 1976 Albany: State University of New York Press

[B93] Strait DL Kraus N Skoe E Ashley R Dalla Bella S, Kraus N, Overy K, Pantev C Musical experience promotes subcortical efficiency in processing emotional vocal sounds, in The neurosciences and music iii: Disorders and plasticity Ann NY Acad Sci 2009 209 13 10.1111/j.1749-6632.2009.04864.x 19673783

[B94] Ohnishi T Matsuda H Asada T Aruga M Hirakata M Nishikawa M Katoh A Imabayashi E Functional anatomy of musical perception in musicians Cereb Cortex 2001 11 754 760 10.1093/cercor/11.8.754 11459765

[B95] Pantev C Oostenveld R Engelien A Ross B Roberts LE Hoke M Increased auditory cortical representation in musicians Nature 1998 392 811 4 10.1038/33918 9572139

[B96] Trainor LJ Desjardins RN Rockel C A comparison of contour and interval processing in musicians and nonmusicians using event-related potentials Australian J Psycholog: Special Issue on Music as a Brain and Behavioural System 1999 51 147 153

[B97] Trainor LJ Are there critical periods for musical development? Dev Psychobiol 2005 46 262 78 10.1002/dev.20059 15772967

